# Leukocyte Immunoglobulin-Like Receptor A3 (LILRA3): A Novel Marker for Lymphoma Development among Patients with Young Onset Sjogren’s Syndrome

**DOI:** 10.3390/jcm10040644

**Published:** 2021-02-08

**Authors:** Evangelia Argyriou, Adrianos Nezos, Petros Roussos, Aliki Venetsanopoulou, Michael Voulgarelis, Kyriaki Boki, Athanasios G. Tzioufas, Haralampos M. Moutsopoulos, Clio P. Mavragani

**Affiliations:** 1 Department of Physiology, Medical School, National and Kapodistrian University of Athens, 11527 Athens, Greece; evargiriou@gmail.com (E.A.); anezos@med.uoa.gr (A.N.); perou89@yahoo.gr (P.R.); 2 Rheumatology Unit, Sismanogleio General Hospital, 15126 Athens, Greece; kboki@outlook.com.gr; 3 Department of Pathophysiology, Medical School, National and Kapodistrian University of Athens, 11527 Athens, Greece; alikivenetsanopoulou@yahoo.com (A.V.); mvoulgar@med.uoa.gr (M.V.); agtzi@med.uoa.gr (A.G.T.); 4 Joint Academic Rheumatology Program, School of Medicine, National and Kapodistrian University of Athens, 11527 Athens, Greece; 5 Athens Academy, Chair Medical Sciences/Immunology, 10679 Athens, Greece; hmoutsop@med.uoa.gr

**Keywords:** LILRA3, Sjogren’s syndrome, lymphoma, chronic inflammation, genes

## Abstract

Background: Primary Sjogren’s syndrome (SS) is an autoimmune disease with a strong predilection for lymphoma development, with earlier disease onset being postulated as an independent risk factor for this complication. Variations of the *Leukocyte immunoglobulin-like receptor A3* *(LILRA3)* gene have been previously shown to increase susceptibility for both SS and non-Hodgkin B-cell lymphoma (B-NHL) in the general population. We aimed to investigate whether variations of the *LILRA3* gene could predispose for lymphoma development in the context of SS. Methods: Study population, all of Greek origin, included 101 SS cases with a current or previous diagnosis of lymphoma (SS-lymphoma, SS-L) and 301 primary SS patients not complicated by lymphoma (SS-non-lymphoma, SS-nL). All SS patients fulfilled the 2016 SS American College of Rheumatology/European league against Rheumatism (ACR/EULAR) classification criteria. A total of 381 healthy controls (HC) of similar age/sex/race distribution were also included. On the basis of the age of SS onset and the presence or absence of adverse predictors for lymphoma development, SS patients were further stratified into younger (≤40 years) and older (>40 years) age of disease onset, as well as into high/medium and low risk groups. Polymerase chain reaction (PCR) was implemented for the detection of the following *LILRA3* gene variants: homozygous non-deleted or functional wild type (+/+) heterozygous (+/−) and homozygous deleted (−/−). LILRA3 serum protein levels were quantitated by enzyme-linked immunosorbent assay (ELISA) in 85 individuals (29 SS-L, 35 SS-nL patients and 21 HC). Results: While no statistically significant differences were detected in the overall frequency of *LILRA3* gene variants between SS-L, SS-nL and HC groups, LILRA3 serum protein levels were increased in the SS-L group compared to HC (1.27 ± 1.34 vs. 0.38 ± 0.34 ng/mL, *p*-value: 0.004). After stratification according to the age of SS onset and history of lymphoma, as well as the presence or absence of adverse predictors for lymphoma development, the prevalence of the functional *LILRA3* gene variant was found to be significantly increased in the young onset SS-L group compared to the HC of similar age and sex distribution (100% vs. 82.9%, *p* = 0.03), as well as in the high/medium risk SS compared to the low risk SS (91.3 vs. 78.3%, *p* = 0.0012). Of note, young onset SS-L and SS-nL groups displayed higher LILRA3 serum levels compared to their older counterparts (*p*-values: 0.007 and 0.0005, respectively). Conclusion: The functional *LILRA3* gene variant increases susceptibility to SS-related lymphoma development in patients with a disease onset of <40 years old, implying that genetically determined deranged immune responses in younger SS individuals could underly their pronounced risk for lymphoma development.

## 1. Introduction

Sjogren’s syndrome (SS) is a chronic autoimmune disease, the second most frequent after rheumatoid arthritis (RA), with an estimated pooled prevalence rate of 60.82 cases per 100,000 inhabitants [1]. Perimenopausal women are mainly affected, with sicca symptoms being the predominant complaints, due to chronically inflamed exocrine glands. Apart from glandular dysfunction, systemic clinical manifestations including musculoskeletal complaints, Raynaud’s phenomenon and peripheral purpura, can also occur [2,3,4]. In a small but significant percentage of SS patients, non-Hodgkin’s lymphoma (NHL) can develop, with the highest likelihood among all autoimmune diseases [5]. Several clinical, serological, histopathological, and genetic contributors have so far been shown to confer increased risk to SS-related lymphoproliferation [6,7]. Deregulated chronic inflammation, B cell hyperactivation, deficiency of immunosurveillance, increased DNA damage and defective DNA methylation have all been proposed as potential pathogenetic determinants [8].

A growing body of data over recent years has supported an emerging role of *Leukocyte immunoglobulin-like receptor subfamily A member 3 (LILRA3)* gene variants in the pathogenesis of both SS and NHL [9,10]. *Leukocyte Immunoglobulin-Like Receptors (LILR)* family is a heterogeneous group of immunoreceptors whose genetic locus is located on chromosome 19 *(19q13.4)*, nearby to the *Killer cell Inhibitory Receptor* (KIR) superfamily. It includes activating (LILRA1, LILRA2, LILRA3) and inhibitory (LIRB1, LILRB2, LILRB3, LILRB4, LILRB5) isoforms [11], depending on their ability to trigger cytokine secretion from monocytes [12] or inhibiting upregulation of co-stimulatory molecules on antigen presenting cells [13,14], respectively. All isoforms are expressed on cells of both myelomonocytic and lymphoid lineage with diverse expression patterns and functional behaviors [11], and have the ability to interact with classical and non-classical Major Histocompatibility Complex class I (MHC-I) antigens [15]. Exceptionally, the *LILRA3* receptor is only encoded in a soluble isoform, lacking a transmembrane or cytoplasmic domain due to alternative mRNA splicing [16]. It is mainly secreted by monocytes and B-cells [17], and induces NK and CD8⁺ T cell proliferation, after allogeneic stimulation [10].

The *LILR* gene cluster consist of two haplotypes, characterized by 13 *LILR* and 12 *LILR* alleles, respectively, as a result of a 6.7 kbp deletion of the *LILRA3* gene. Particularly, the shorter haplotype is characterized by the absence of the first seven of its eight exons [18]. Of interest *LILRA3* variants have shown great heterogeneity across races, with the *LILRA3* deleted variant being the dominant allele in northeast Asia (~84%), and the functional genotype (*LILRA3+/+)* being dominant in Caucasian populations [19]. In a German cohort, the presence of the deleted form has been associated with SS [9] and NHL susceptibility [10], while in a Chinese population, the presence of the functional *LILRA3* variant increased disease susceptibility for both SS and active systemic lupus erythematosus (SLE), along with the presence of serum anti-Ro/SSA and anti-La/SSB antibodies and leucopenia [20,21], both previously shown to serve as high risk predictors for SS related lymphoma [22,23].

In the current study, we aimed to investigate whether *LILRA3* gene polymorphisms and LILRA3 serum levels increase susceptibility to SS-related lymphoma in a Greek SS cohort.

## 2. Patients and Methods

### 2.1. Study Participants

The present case control study included 101 SS-Lymphoma (SS-L) cases (92.1% females, mean age ± SD: 51.2 ± 13.3 years) with a presence or history of lymphoma and 301 primary SS patients not complicated by lymphoma (SS-non-lymphoma, SS-nL) (92.7% females, mean age ± SD: 52.4 ± 13.8 years). All SS patients fulfilled the 2016 SS American College of Rheumatology/European league against Rheumatism (ACR/EULAR) classification criteria [24]. A total of 381 healthy controls (HC) of similar age/sex/race distribution (89.2% females, mean age ± SD: 57.1 ± 17.3 years) were also included. All study participants were of Caucasian origin. None of the controls had a current or previous diagnosis of lymphoma. On the basis of the age of SS onset, SS patients were further stratified into younger (≤40 years) and older (>40 years) disease onset groups. Moreover, on the basis of the number of adverse predictors present at the time of diagnosis (salivary gland enlargement (SGE), lymphadenopathy, Raynaud’s phenomenon, anti-Ro/SSA or/and anti-La/SSB autoantibodies, rheumatoid factor (RF) positivity, monoclonal gammopathy, and C4 hypocomplementemia), SS-nL patients were further classified into low risk (≤2 adverse predictors) and medium/high risk for lymphoma development (3–7 adverse predictors), as previously described [22]. Additionally, a subgroup of SS patients was further classified according to the age of disease onset (defined as the date of disease diagnosis) into the following groups: SS-nL patients ≤40 years (*n* = 52), SS-L patients ≤40 years (*n* = 23) and SS-nL patients >40 years (*n* = 249), SS-L patients >40 years (*n* = 78). Demographic, clinical and laboratory characteristics were recorded in all study participants, as previously described [25]. Whole blood samples were collected from study populations and stored in Ethylene Diamine Tetraacetic Acid (EDTA) tubes at −20 °C.

### 2.2. DNA Extraction

DNA isolation was performed using Macherey-Nagel Nucleospin Blood Kit (Macherey-Nagel, Düren, Germany) according to the manufacturer’s instructions. Spectophotometry (Biospec Nano, Japan) was utilized to confirm the quantity and quality of DNA samples.

### 2.3. Polymerase Chain Reaction (PCR)/LILRA3 Gene Polymorphisms

The detection of *LILRA3* gene polymorphisms was performed by PCR, according to the previously reported method [26] (See Supplementary. Methods for further details). We identified three genetic variations: homozygous non-deleted (functional or wild type, +/+), heterozygous (+/−) and homozygous deleted (−/−).

### 2.4. Enzyme-Linked Immunosorbent Assay (ELISA)

Quantification of LILRA3 protein levels was performed using LILRA3 ELISA kit (Cusabio Technology LLC. Wuhan, China, *Catalogue Number, CSB-EL012935HU*) in 85 available serum samples from 29 SS-L patients (16 SS-L ≤40 years, 13 SS-L >40 years), 35 SS-nL patients (20 SS-nL≤40 years, 15 SS-nL > 40 years) and 21 HC (12 ≤40 years and 9 > 40 years old). According to the manufacturer’s instructions, LILRA3 ELISA detects soluble LILRA3 levels, which are encoded by the functional *LILRA3* gene.

### 2.5. Statistical Analysis

All statistical analyses were performed using SPSS v.25.0 (IBM, Armonk, NY, U.S.) and GraphPad Prism 8 (GraphPad Software, San Diego, CA, U.S.), with the level of statistical significance being set at 0.05. Chi square or Fisher’s exact test was performed to compare the frequencies of deleted and non-deleted gene variants among SS patients and healthy population. Mann–Whitney test was employed for detecting the difference in serum LILRA3 protein levels among study participants.

## 3. Results

### 3.1. Prevalence of LILRA3 Genetic Variants in SS Patients and HC

**Figure 1:**
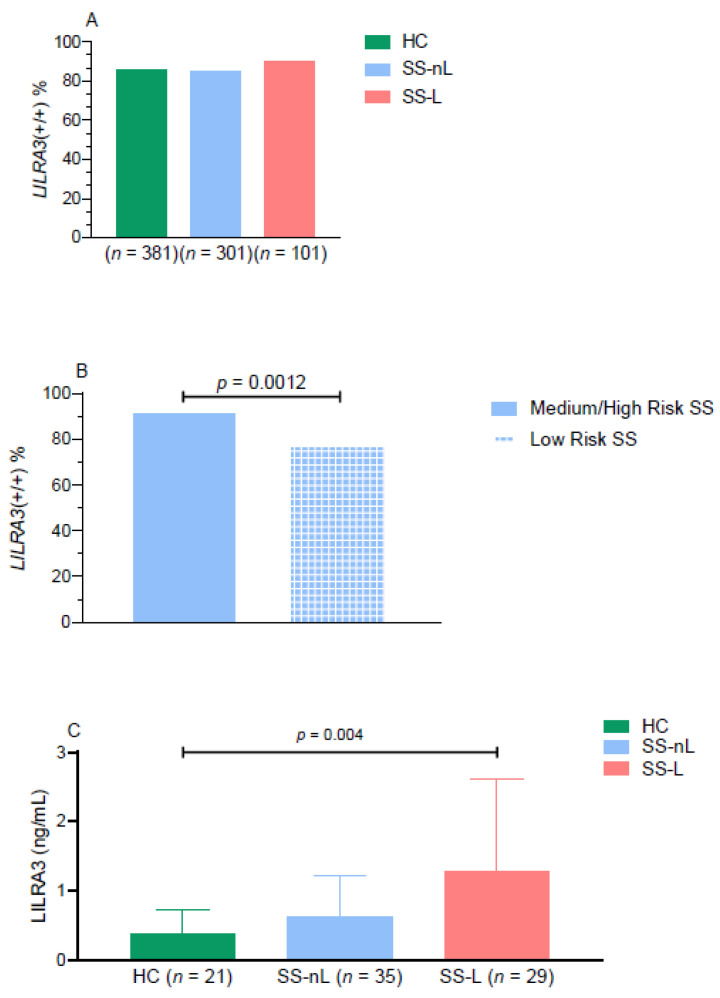
(**A**) Frequency of the *LILRA3* wild type variant among distinct SS subsets in comparison to HC. (**B**) Statistically increased frequency of wild type *LILRA3* was detected in medium/high risk group compared to low risk SS patients. Stratification of SS patients was based on the number of adverse predictors for lymphoma development at diagnosis (≤2 risk factors: low risk, >2 risk factors: medium/high risk. (**C**) Serum levels of LILRA3 protein in distinct SS subsets and HC. LILRA3: leukocyte immunoglobulin-like receptor A3; SS-nL: Sjogren’s syndrome non-lymphoma patients; SS-L: Sjogren’s syndrome lymphoma patients; HC: healthy controls.

As displayed in Table 1, there was no statistically significant differences in the frequency of *LILRA3* genotypes between HC and SS subpopulations (SS-nL/SS-L) (Figure 1A). Of interest, after we stratified SS-nL patients into high/medium and low risk based on the number of adverse predictors present at disease diagnosis, the *LILRA3* functional variant was detected in 91.3% of high/medium risk SS-nL patients compared to 78.3% in the low risk SS-nL group (*p* = 0.0012, Figure 1B).

**Figure 2:**
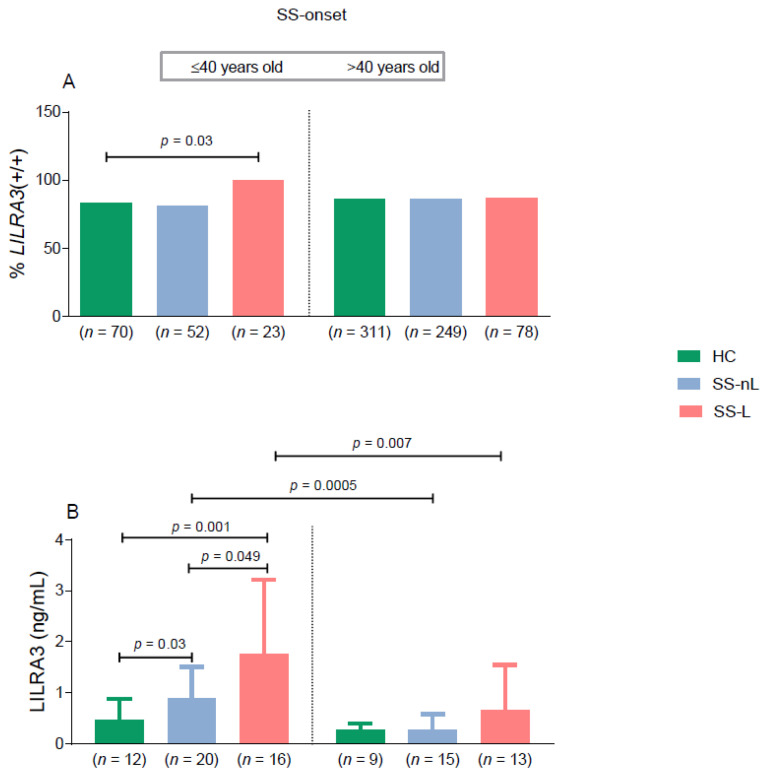
(**A**) Prevalence of the *LILRA3* functional (wild) type variant *(LILRA3+/+)* in HC and distinct SS subgroups stratified by age of SS onset and lymphoma history. Statistically significant increased prevalence of the wild type *LILRA3* variant in the SS-L subgroup with SS onset ≤40 years compared to HC. No statistically significant difference was detected in the frequency of the wild type variant among SS subgroups with SS onset >40 years and HC. (**B**) LILRA3 serum protein levels in SS patients and HC stratified by age of disease onset and the presence of lymphoma history. Statistically significant increased levels of LILRA3 protein serum levels were mainly observed in SS-L patients ≤40 years compared to HC. LILRA3: leukocyte immunoglobulin-like receptor A3; SS-nL: Sjogren’s syndrome non-lymphoma patients; SS-L: Sjogren’s syndrome lymphoma patients; HC: healthy controls. Only significant comparisons are shown.

Given our previous observations supporting an accumulation of genetic abnormalities in the younger onset SS subgroup complicated by lymphoma [27,28], we wished to explore whether SS patients with younger disease onset (≤40 years old) also displayed higher frequencies of *LILRA3* variations. Indeed, after SS patients were stratified according to the age of disease onset, the SS-L, but not the SS-nL group with disease onset ≤ 40 years displayed statistically significant higher rates of the wild type variant of the *LILRA3* gene compared to HC of similar age and sex distribution (SS-L: 100%, HC: 82.9%, *p* = 0.03). No differences were detected in the prevalence of the *LILRA3+/+* genotype between SS subgroups with disease onset >40 years old and HC (SS-L: 87.2%, SS-nL: 85.9%, HC: 86.2%, all comparisons non-significant, Figure 2A).

Following classification according to lymphoma subtype, the prevalence of the functional *LILRA3*+/+ gene was 88.9% in 18 non-marginal zone lymphoma cases (13 with diffuse large B-cell lymphoma, two with small lymphocytic lymphoma, one with follicular lymphoma, one with nodular lymphoma and one with T-cell lymphoma), 88.7% in marginal zone lymphoma localized in the salivary glands (*n* = 71) and 100% in SS- disseminated marginal zone lymphoma (*n* = 12) (Supplementary Figure S1).

### 3.2. LILRA3 Serum Protein Levels

Given that LILRA3 serum protein levels were previously shown to be elevated in patients with chronic inflammatory diseases [17,29], we next aimed to explore whether they are also increased in SS. As displayed in Figure 1C, LILRA3 serum protein levels were significantly increased only in the SS-L compared to the HC group (1.27 ± 1.34 ng/mL vs. 0.38 ± 0.34 ng/mL, *p*-value: 0.004), with no statistically significant differences detected between SS-nL compared to SS-L and HC subsets (0.63 ± 0.59 ng/mL vs. 1.27 ± 1.34 ng/mL and 0.38 ± 0.34 ng/mL, *p*-values 0.06 and 0.16, respectively). After stratification according to the age of SS onset and history of lymphoma (Figure 2B), younger onset SS-L and SS-nL patients displayed significantly higher serum LILRA3 protein levels compared to HC (1.77 ± 1.45 ng/mL and 0.89 ± 0.61 ng/mL vs. 0.46 ± 0.43 ng/mL, *p*-values 0.001 and 0.03, respectively). LILRA3 serum levels were also increased in SS-L compared to SS-nL patients of young onset (*p*-value 0.049). No significant differences were detected in serum LILRA3 levels between SS groups with disease onset more than 40 years compared to HC (SS-L: 0.66 ± 0.89 ng/mL, SS-nL: 0.28 ± 0.30 ng/mL, HC: 0.27 ± 0.14 ng/mL). Of interest, LILRA3 serum levels were significantly increased in both SS-L and SS-nL groups of younger onset compared to those with disease onset later in life (*p*-values 0.007 and 0.0005, respectively). No such differences were found between HC following age stratification. Finally, there was a trend towards increased LILRA3 serum levels in medium/high risk SS patients compared to low risk, though the difference did not reach statistical significance (low risk: 0.40 ± 0.42 ng/mL, medium/high risk: 0.70 ± 0.85 ng/mL) (Supplementary Figure S2).

### 3.3. LILRA3 Functional Variant-Clinical and Laboratory Associations

As shown in Supplementary Table S1, among SS-L patients, those bearing the functional *LILRA3 +/+* variant demonstrated increased prevalence of anti-Ro/SSA and/or anti-La/SSB antibodies compared to those with the homozygous or heterozygous deletion (89% vs. 60%, *p* = 0.03, respectively), as well as a trend towards increased LILRA3 serum levels among all SS patients bearing the wild type polymorphism (Supplementary Figure S3). No other significant associations of *LILRA3* gene variants with demographics, clinical or laboratory characteristics were detected in either SS-L or SS-nL groups.

## 4. Discussion

To our knowledge, this is the first study revealing *LILRA3* gene variants and the corresponding LILRA3 serum levels to be associated with increased lymphoproliferative risk in the setting of a young onset Greek-SS population. Indeed, the frequency of the functional *LILRA3 (+/+)* genotype was found to be significantly increased in SS-L patients with SS diagnosis ≤40 years old compared to healthy controls of similar age and sex distribution. Moreover, LILRA3 serum protein levels were significantly higher in young onset SS patients compared to both young onset SS patients with no lymphoma and healthy controls. Of interest, SS patients at high/medium risk for lymphoma development also displayed increased frequency of the *LILRA3 (+/+)* genotype compared to the low risk group. Finally, LILRA3 serum levels were significantly increased in both SS-L and SS-nL groups of younger onset compared to those with disease onset later in life.

The association of the *LILRA3* functional variant with lymphoma development in the context of younger onset SS patients, together with the heightened LILRA3 serum levels in SS individuals with a younger age of disease onset (≤40 years old), reinforce our previous observations at both clinical and genetic grounds. Thus, a growing body of evidence supports an aggressive phenotype among SS patients with younger disease onset, indicating strong immunological activity and a highly inflammatory background [30,31,32,33,34]. Moreover, the rs2230926 exonic variant of the *Tumor Necrosis Factor, Alpha-Induced Protein 3* (TNFAIP3) gene, encoding a defective A20 protein, along with the *BAFF-R His159Tyr* mutation, have been detected in increased frequency among young SS patients (≤40 years) complicated with lymphoma [27,28]. Both mutations have been shown to be linked with NF-κB hyperactivation, triggering an ongoing inflammatory response [35], already proposed as a central pathogenetic event in SS related lymphoma.

Given that the LILRA3 protein has been previously found to be elevated in both sera from patients with RA, SLE and multiple sclerosis (MS) in association with disease activity [17,21] or severity [29], it seems that the LILRA3 molecule may contribute to SS and SS related lymphoma development in younger onset populations through altered regulation of chronic inflammatory processes. In support of this hypothesis, the LILRA3 protein has been previously shown to trigger a cellular immune response, through proliferation of cytotoxic-CD8⁺T and NK cells [10], both previously shown to be related to SS pathogenesis [36]. Moreover, LILRA3 molecule has a beneficial effect on clearing HIV virus, potentially through induction of proinflammatory cytokines [37], though there are data supporting an anti-inflammatory action as well [17,29,38].

The findings of the present study are somewhat intriguing. Although the Caucasian pattern regarding the prevalence of *LILRA3* gene was observed in the Greek cohort, the contribution of the wild rather than the deleted form of the *LILRA3* gene seems to increase susceptibility to autoimmunity. Thus, in accord with our findings, the functional rather than the deleted variant has been linked to several autoimmune disorders in Asian populations such as RA [39], ankylosing spondylitis [40], SLE, and SS [20]. Moreover, in Chinese populations, it was the functional allele which was found to serve as risk factor for seropositive SS [20]. Given that autoantibodies against Ro/SSA and La/SSB autoantigens have been previously designated as risk factors for lymphoma development [22], the association of the functional variant with SS related lymphoma in our study seems to be compatible with these findings. It is unclear at this point why in other Caucasian populations, such as German [9,41] or Spanish [26] populations, the *LILRA3* deletion has been identified as a risk factor for SS and MS.

Although the study includes complete evidence about the clinical, serological, and immunological profile of the SS patients, the size of the SS-subgroups is limited for extracting more robust data, mainly due to the rarity of this SS complication, particularly among patients with young onset disease. Last, further functional characterization of the LILRA3 molecule is mandatory to explore how chronic immune responses result in oncogenic events.

Taken together, these findings support a potential role of the LILRA3 molecule in the pathogenesis of SS related lymphoma, giving an insight into the aggressive nature of young onset SS, characterized by profound B cell activation. Further studies in larger populations are needed to confirm these findings and clarify the contribution of LILRA3 in promoting chronic inflammatory pathways in the setting of autoimmunity and lymphoproliferation.

## Figures and Tables

**Table 1:** Frequency of *LILRA3* genotypes in distinct SS subpopulations and HC.

Prevalence of *LILRA3* Variants			
*LILRA3* Genotype (*n*, %)	HC (*n* = 381)	SS-nL (*n* = 301)	SS-L (*n* = 101)
(+/+)	326 (85.6%)	256 (85.0%)	91 (90.1%)
(+/−)	54 (14.2%)	44 (14.7%)	9 (8.9%)
(−/−)	1 (0.2%)	1 (0.3%)	1 (1%)

LILRA3: leukocyte immunoglobulin-like receptor A3; SS-nL: Sjogren’s syndrome non-lymphoma patients; SS-L: Sjogren’s syndrome lymphoma patients; HC: healthy controls.

## Data Availability

we declare that data are available on request due to confidentiality of personal information.

## Supplementary Materials

The Supplementary Material for this article can be found online at https://www.mdpi.com/2077-0383/10/4/644/s1, Table S1: Prevalence of *LILRA3* genetic variants in distinct SS subpopulations along with clinical and laboratory associations, Figure S1: Increased prevalence of the *LILRA3*+/+ variant in SS patients with marginal zone disseminated B-cell lymphoma, though differences did not reach statistical significance, Figure S2: Increased LILRA3 serum levels in medium/high risk SS patients compared to the low risk SS group, Figure S3: Increased LILRA3 serum levels in patients with functional *LILRA3* variant (+/+) compared to those with *LILRA3* (+/− or −/−) variants.
