# Supplementary material for: Leukocyte Immunoglobulin-Like Receptor A3 (LILRA3): A Novel Marker for Lymphoma Development among Patients with Young Onset Sjogren’s Syndrome
Source: J Clin Med. 2021 Feb 8;10(4):644. doi: 10.3390/jcm10040644 (PMC7915360; doi:10.3390/jcm10040644)

Suppl. Methods

The detection of LILRA3 gene polymorphisms was performed with PCR method, using for each sample in the same tube the following triad of primers: Forward 1 (FW1): *5′-gacttgtaagggttaaaaagccaa-3′/* Forward 2 (FW2): *5′-catctcgatctgccactgacac-3′/* REVERSE(R): *5′-gacagcagattctaaaacagtgg-3′.* The product size resulting from combination of FW1-R is 150 bp, representative of the complete LILRA3 gene, while the combination of FW2-R primers results in a 241 bp product, not including FW1-R target site, leading to LILRA3 deletion (absence of first seven exons).

Suppl. Table S1. Prevalence of LILRA3 genetic variants in distinct SS subpopulations along with clinical and laboratory associations. LILRA3: Leukocyte immunoglobulin-like receptor A3, SS-nL: Sjogren's Syndrome non-Lymphoma patients, SS-L: Sjogren's Syndrome Lymphoma patients, SGE: Salivary gland enlargement, RF: Rheumatoid factor, WBC: White blood cells

	SS-nL (n= 301)			SS-L (n= 101)		
**LILRA3 genetic variants n (%)**	**LILRA3 +/+** 256 (85.0)	**LILRA3+/- or LILRA3 -/-** 45 (15)	**p-value**	**LILRA3 +/+** 91 (90)	**LILRA3+/- or LILRA3 -/-** 10 (10)	**p-value**
**Demographics**						
Age of sample (years, mean±SD)	58.3 ± 13.6	57.6 ± 14.2	NS	59.0 ± 12.6	63.3 ± 11.3	NS
Sex (% female)	93	91.1	NS	93.4	80	NS
**Glandular manifestations**						
Dry mouth subjective (%)	91.7	3.3	NS	98.9	100	NS
Dry eyes subjective (%)	88.2	86.7	NS	93.4	100	NS
SGE (%)	22.1	15.6	NS	65.6	80	NS
Positive Schirmer's test (%)	75.8	70.4	NS	92	100	NS
No of lymphocytic infiltrations per $4mm^2 \geq 1$ (%)	59.6	54.1	NS	78.4	77.8	NS
**Systemic manifestations**						
Arthritis (%)	21.6	22.2	NS	17.6	40	NS
Raynaud's phenomenon (%)	26.7	22.2	NS	30.8	10	NS
Purpura	11.4	8.9	NS	34.1	30	NS
**Laboratory features**						
WBC<3000 $/mm^3$	2.8	0	NS	3.4	0	NS
Anti- Ro/SSA and/ or La/SSB (%)	70.8	65.9	NS	89	60	0.03
Positive RF titers (>20IU/ml)	52	54.8	NS	83.7	80	NS
C4≤20 mg/dl	49.4	44.2	NS	79.3	66.7	NS
Monoclonal gammopathy	5.7	5	NS	19.8	20	NS
Cryoglobulins	5.5	9.3	NS	41.8	22.2	NS

Suppl. Fig.S1. Increased prevalence of the LILRA3+/+ variant in SS patients with marginal zone disseminated B-cell lymphoma, though differences did not reach statistical significance

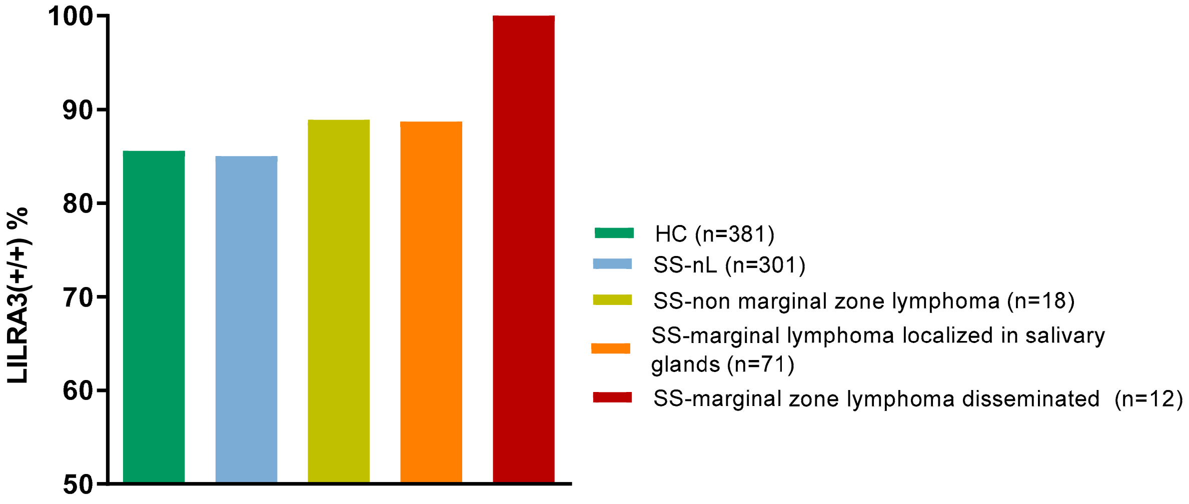

Suppl. Fig. S2. Increased LILRA3 serum levels in medium/high risk SS patients compared to the low risk SS group. The difference did not reach statistical significance.

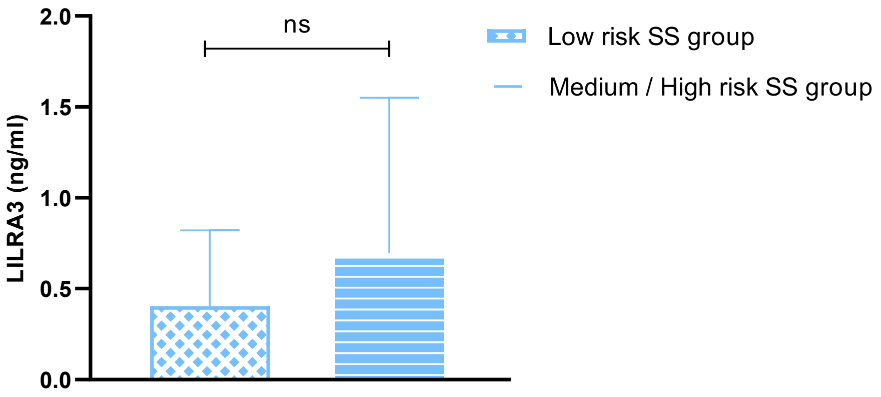

Suppl. Fig. S3. Increased LILRA3 serum levels in patients with functional LILRA3 variant (+/+) compared to those with LILRA3 (+/- or -/-) variants.

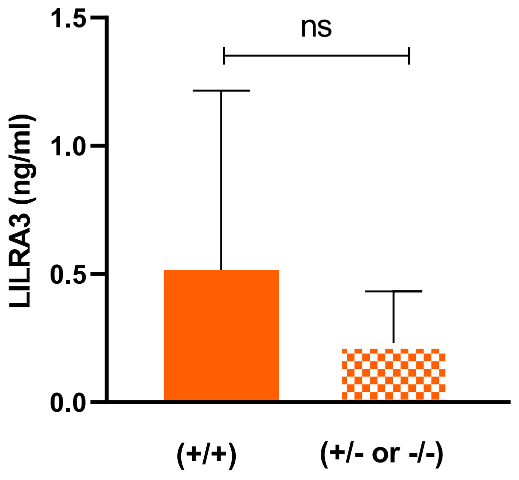

Supplement: Supplementary file 1 [file jcm-10-00644-s001.pdf]
